# Supplementary material for: NMNAT2 supports vesicular glycolysis via NAD homeostasis to fuel fast axonal transport
Source: Mol Neurodegener. 2024 Jan 29;19:13. doi: 10.1186/s13024-023-00690-9 (PMC10823734; doi:10.1186/s13024-023-00690-9)
Supplement: Supplementary file 1 — Additional file 1: Fig. 1-S1. NMNAT2 expression, cKO mice survival rate, and motor behavioral deficit (A) In situ hybridization reveals nmnat2 mRNAs (blue signal) in the E14.5 brain, mainly in the cortical plate (CP) and in the lateral ganglionic eminence (LGE) and medial ganglionic eminence (MGE). (B) Illustration of Nmnat2 conditional KO (cKO) mice generation as described previously [37]. The blue and magenta triangles indicate loxP sites. (C, D) Summary of embryonic and postnatal pups genotyping. For embryonic samples, the strategy was to breed Nex-Cre/+;NMNAT2f/+ with NMNAT2f/+ mice. The genotyping results conform to the expectations of Mendelian inheritance. The breeding strategy for postnatal pups was to cross Nex-Cre/+;NMNAT2f/+ with NMNAT2f/f mice. The genotyping result showed a smaller than expected number of mice with the Nex-Cre/+;NMNAT2f/f (cKO) genotype, suggesting a reduced survival rate for cKO mice after birth. (E) Overall numbers of postnatal male and female ctrl and cKO mice. (F) 3-5 months old NMNAT2 cKO mice exhibit evident hindlimb clasping behavior (ctrl N=3, cKO N=4 were scored), Mann-Whitney test was used, data represents mean ± SEM, *p=0.04. Abbreviations: Cre, Nex-Cre; f/f, NMNAT2f/f; IZ, intermediate zone; Str, striatum; SVZ, subventricular zone; VC, ventricle; VZ, ventricular. Fig. 2-S1. Loss of NMNAT2 triggers APP accumulation in the axon of cultured cortical neurons (A) Representative confocal image of APP immunocytochemical staining in ctrl and KO neuronal culture at DIV5, 8, 10, and 14. Scale bar 30 μm. Red arrows highlight APP accumulation. (B) Area of accumulated APP in cKO and ctrl mice normalized to the average ctrl value of the corresponding DIV. Image numbers used for analysis: DIV5: 10 ctrl, 9 KO; DIV8 & DIV10: 20 ctrl, 20 KO; DIV14: 50 ctrl, 67 KO. DIV5-10 were from 2 independent culture experiments and DIV14 was from > 3 independent experiments. Multiple Mann-Whitney test with Holm-Šídák post-hoc adjustment, KO was compared t [file 13024_2023_690_MOESM1_ESM.pdf]

Fig 1-S1

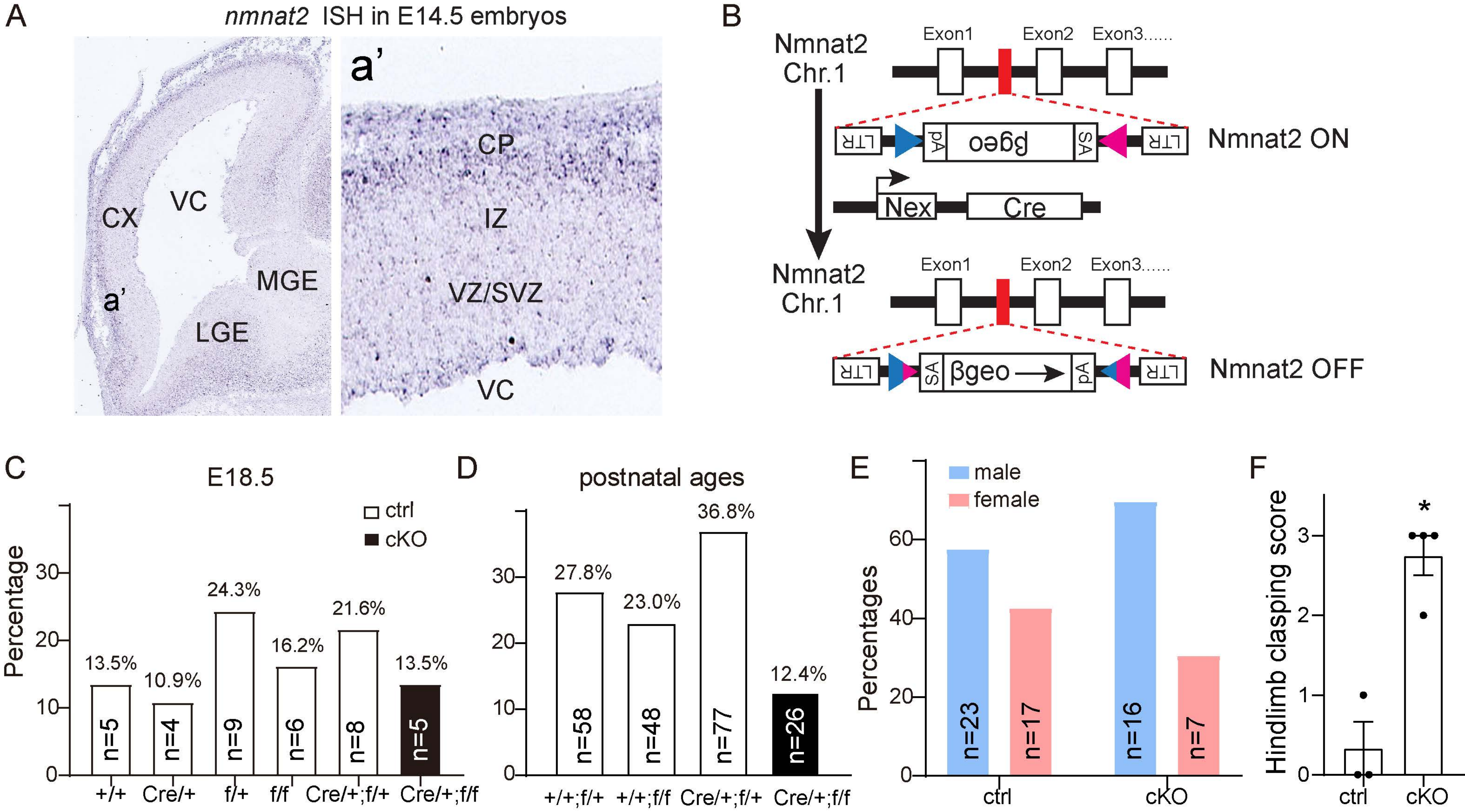
A
nmnat2 ISH in E14.5 embryos
CX
VC
a'
MGE
LGE
CP
IZ
VZ/SVZ
VC
B
Nmnat2 Chr.1
Exon1
Exon2
Exon3......
LTR
pA
βgeo
SA
LTR
Nmnat2 ON
Nex
Cre
Nmnat2 Chr.1
Exon1
Exon2
Exon3......
LTR
SA
βgeo
pA
LTR
Nmnat2 OFF
C
E18.5
ctrl
cKO
Percentage
0
10
20
30
13.5%
10.9%
24.3%
16.2%
21.6%
13.5%
n=5
n=4
n=9
n=6
n=8
n=5
+/+
Cre/+
f/+
f/f
Cre/+;f/+
Cre/+;f/f
D
postnatal ages
0
10
20
30
27.8%
23.0%
36.8%
12.4%
n=58
n=48
n=77
n=26
+/+;f/+
+/+;f/f
Cre/+;f/+
Cre/+;f/f
E
male
female
Percentages
0
20
40
60
n=23
n=17
n=16
n=7
ctrl
cKO
F
Hindlimb clasping score
0
1
2
3
*
ctrl
cKO

Fig. 2-S1

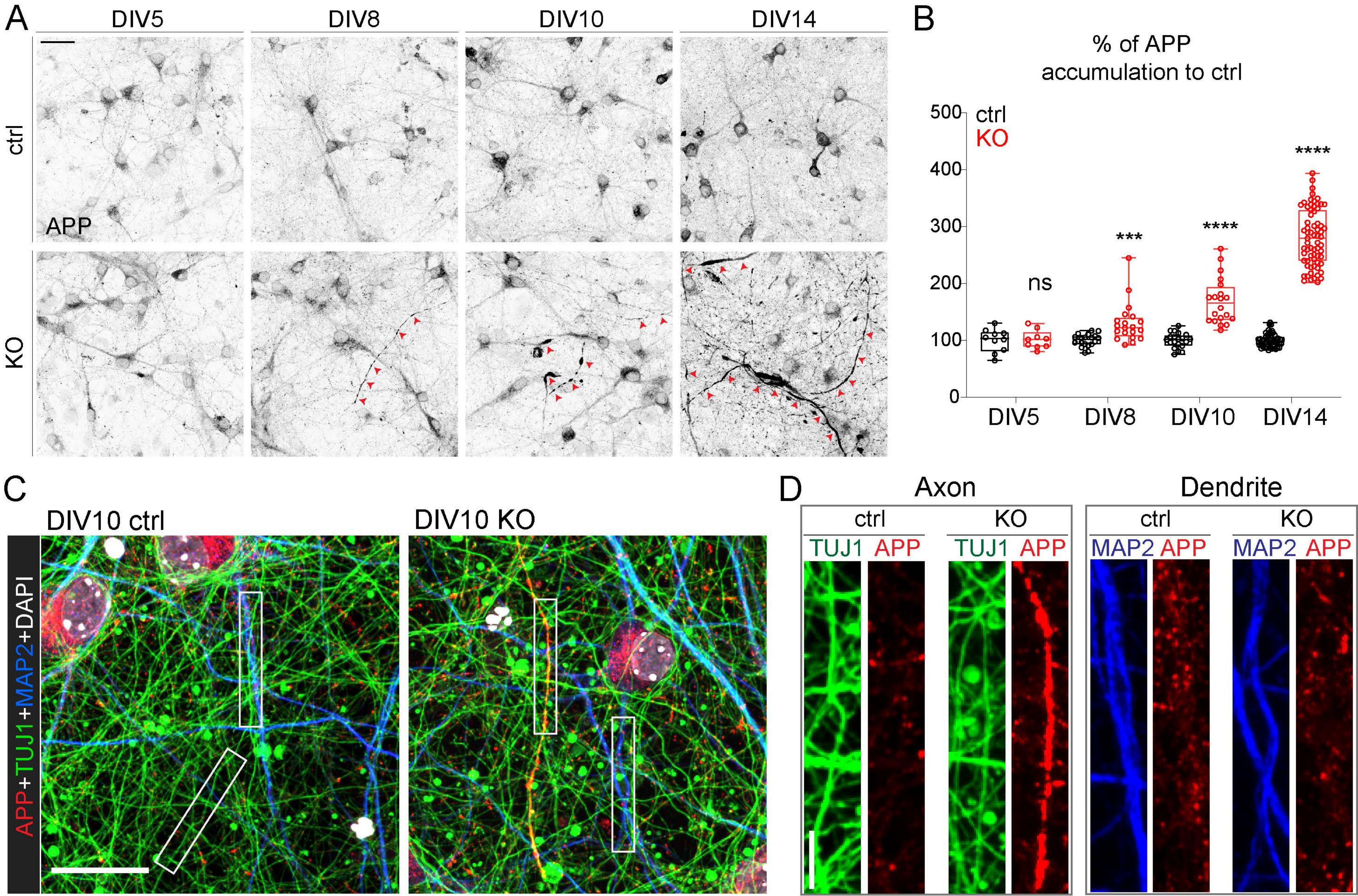
A
DIV5
DIV8
DIV10
DIV14
ctrl
KO
APP
B
% of APP
accumulation to ctrl
ctrl
KO
500
400
300
200
100
0
ns
***
****
****
DIV5
DIV8
DIV10
DIV14
C
DIV10 ctrl
DIV10 KO
APP+TUJ1+MAP2+DAPI
D
Axon
Dendrite
ctrl
KO
TUJ1 APP
TUJ1 APP
ctrl
KO
MAP2 APP
MAP2 APP

Fig. 2-S2

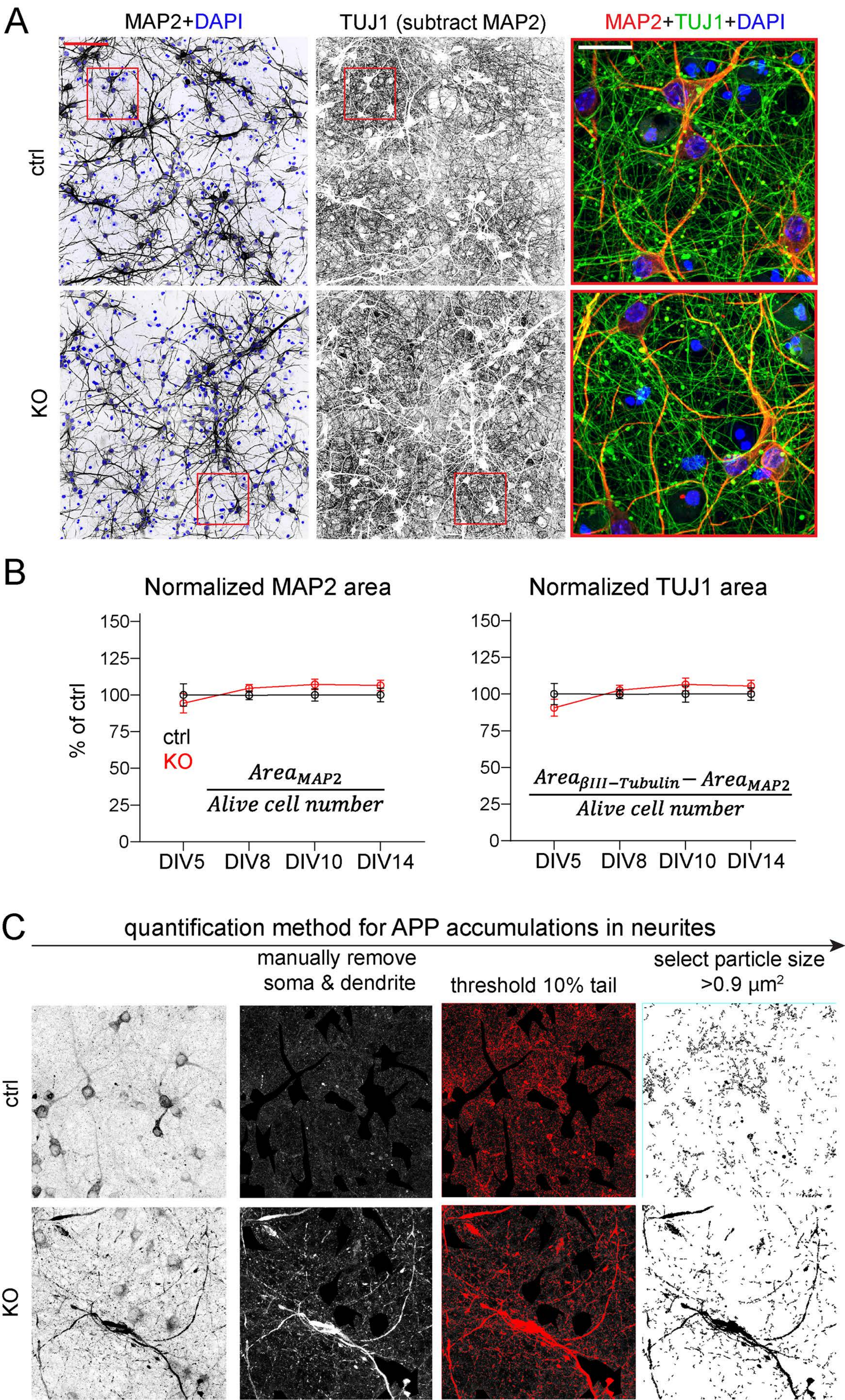
A
MAP2+DAPI
TUJ1 (subtract MAP2)
MAP2+TUJ1+DAPI
ctrl
KO
B
Normalized MAP2 area
Normalized TUJ1 area
% of ctrl
150
125
100
75
50
25
0
ctrl
KO
$\frac{Area_{MAP2}}{Alive\ cell\ number}$
$\frac{Area_{\beta III-Tubulin} - Area_{MAP2}}{Alive\ cell\ number}$
DIV5
DIV8
DIV10
DIV14
C
quantification method for APP accumulations in neurites
manually remove soma & dendrite
threshold 10% tail
select particle size >0.9 μm²
ctrl
KO

Fig. 2-S3

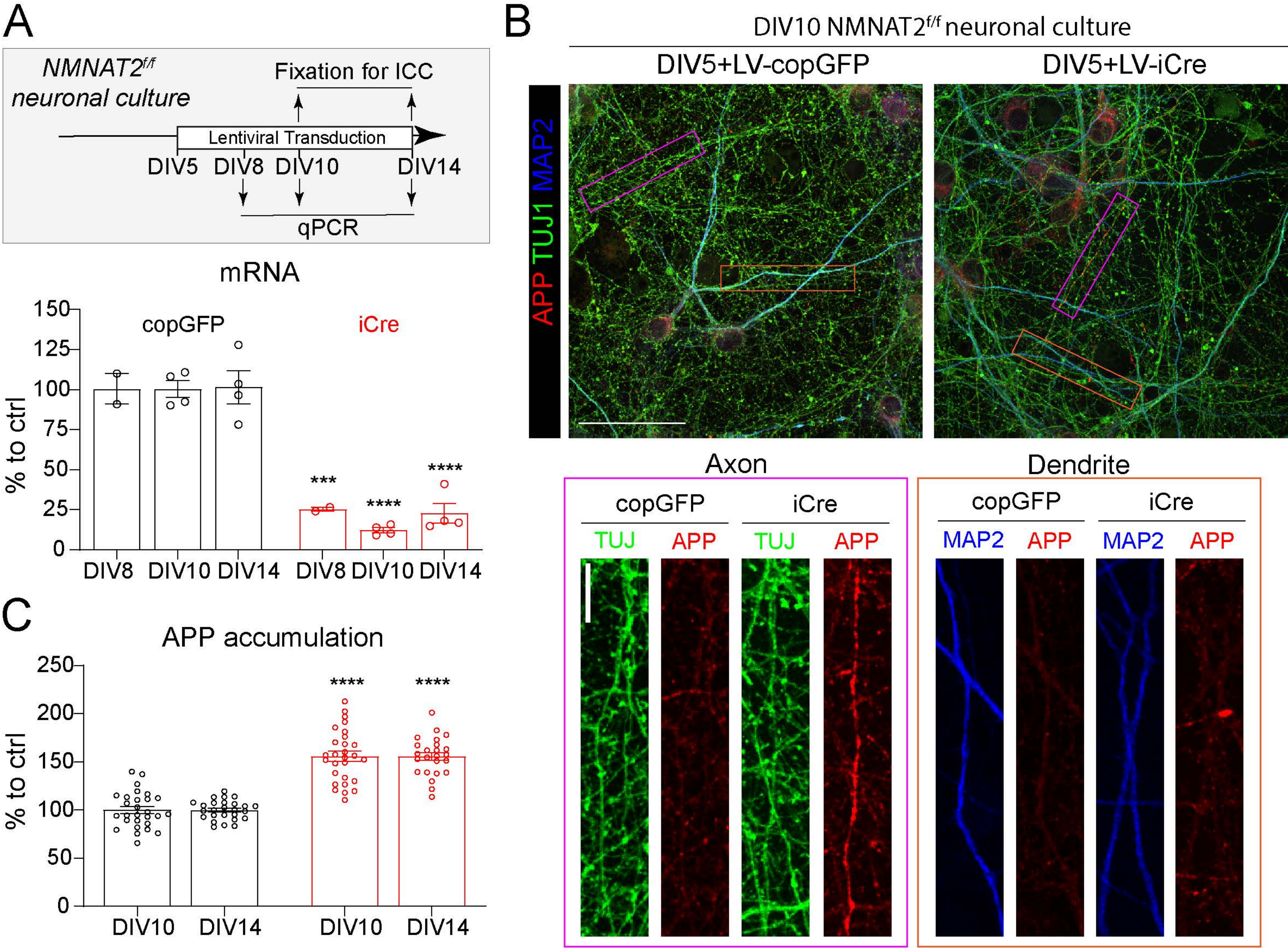

A
NMNAT2f/f neuronal culture
Fixation for ICC
Lentiviral Transduction
DIV5 DIV8 DIV10 DIV14
qPCR
mRNA
copGFP
iCre
% to ctrl
150
125
100
75
50
25
0
***
****
****
DIV8 DIV10 DIV14 DIV8 DIV10 DIV14
C
APP accumulation
% to ctrl
250
200
150
100
50
0
****
****
DIV10 DIV14 DIV10 DIV14
B
DIV10 NMNAT2f/f neuronal culture
DIV5+LV-copGFP
DIV5+LV-iCre
APP TUJ1 MAP2
Axon
Dendrite
copGFP
iCre
TUJ
APP
MAP2

Fig 3-S1

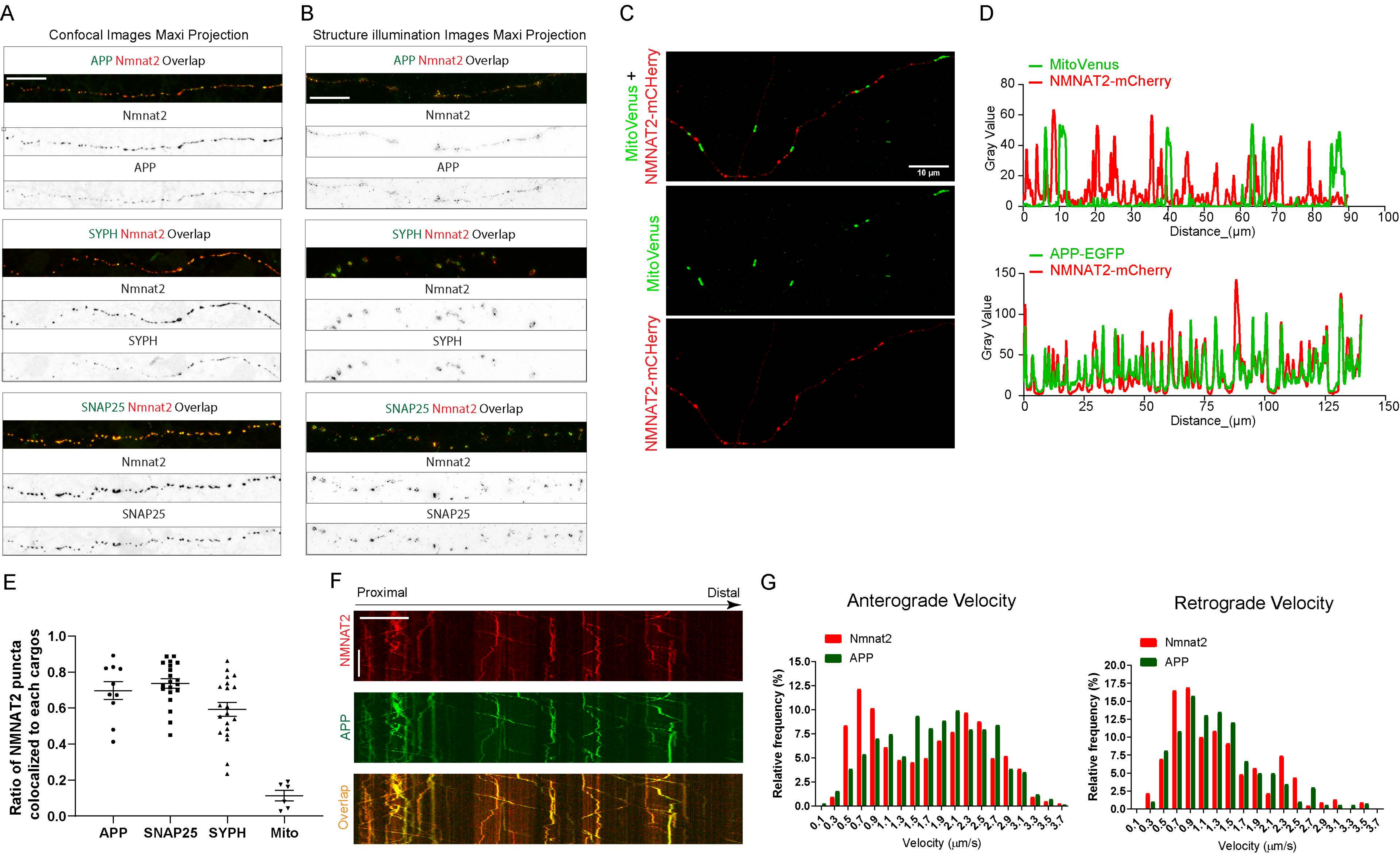

A
Confocal Images Maxi Projection
APP Nmnat2 Overlap
Nmnat2
APP
SYPH Nmnat2 Overlap
Nmnat2
SYPH
SNAP25 Nmnat2 Overlap
Nmnat2
SNAP25
B
Structure illumination Images Maxi Projection
APP Nmnat2 Overlap
Nmnat2
APP
SYPH Nmnat2 Overlap
Nmnat2
SYPH
SNAP25 Nmnat2 Overlap
Nmnat2
SNAP25
C
MitoVenus + NMNAT2-mCHerry
10 µm
MitoVenus
NMNAT2-mCHerry
D
MitoVenus
NMNAT2-mCherry
Gray Value
Distance_(µm)
APP-EGFP
NMNAT2-mCherry
Gray Value
Distance_(µm)
E
Ratio of NMNAT2 puncta colocalized to each cargos
APP
SNAP25
SYPH
Mito
F
Proximal
Distal
NMNAT2
APP
Overlap
G
Anterograde Velocity
Nmnat2
APP
Relative frequency (%)
Velocity (µm/s)
Retrograde Velocity
Nmnat2
APP
Relative frequency (%)
Velocity (µm/s)

Fig. 3-S2

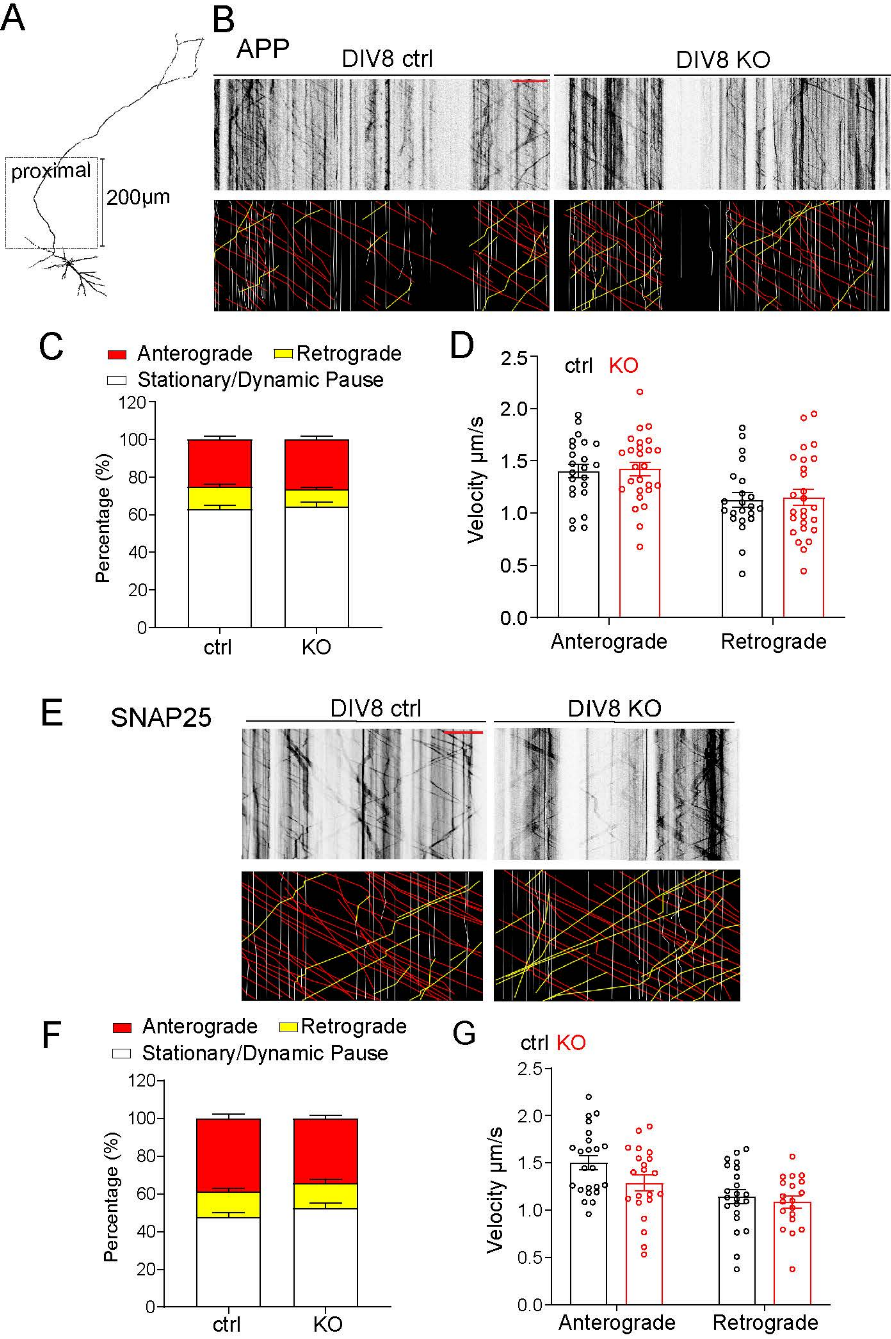
A
proximal
200μm
B
APP
DIV8 ctrl
DIV8 KO
C
Anterograde
Retrograde
Stationary/Dynamic Pause
Percentage (%)
120
100
80
60
40
20
0
ctrl
KO
D
ctrl
KO
Velocity μm/s
2.5
2.0
1.5
1.0
0.5
0.0
Anterograde
Retrograde
E
SNAP25
DIV8 ctrl
DIV8 KO
F
Anterograde
Retrograde
Stationary/Dynamic Pause
Percentage (%)
120
100
80
60
40
20
0
ctrl
KO
G
ctrl
KO
Velocity μm/s
2.5
2.0
1.5
1.0
0.5
0.0
Anterograde
Retrograde

Fig 3-S3

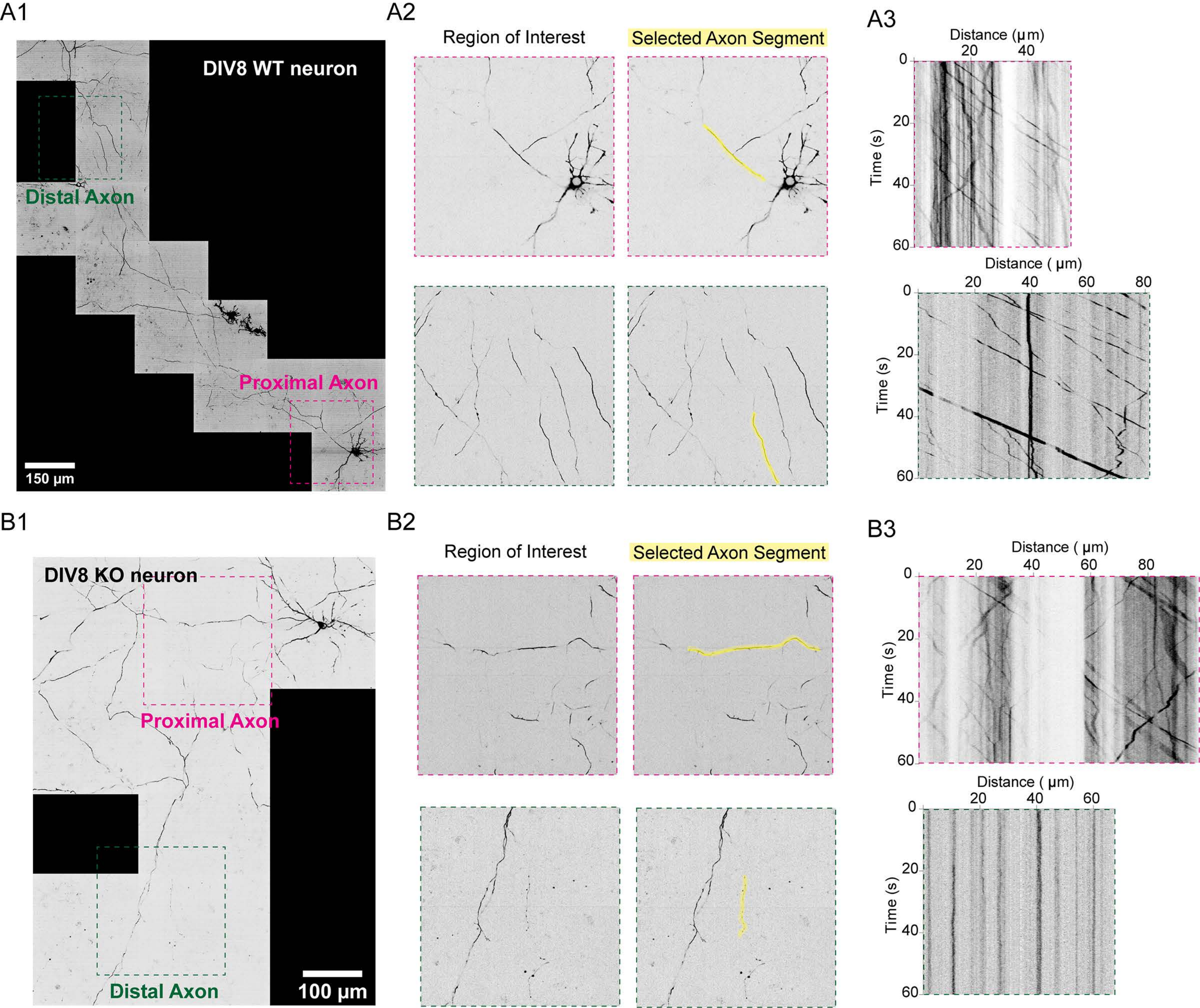

A1
DIV8 WT neuron
Distal Axon
Proximal Axon
150 μm
A2
Region of Interest
Selected Axon Segment
A3
Distance (μm)
20
40
0
20
40
60
Time (s)
Distance (μm)
20
40
60
80
B1
DIV8 KO neuron
Proximal Axon
Distal Axon
100 μm
B2
Region of Interest
Selected Axon Segment
B3
Distance (μm)
20
40
60
80
Time (s)
Distance (μm)
20
40
60

Fig 3-S4

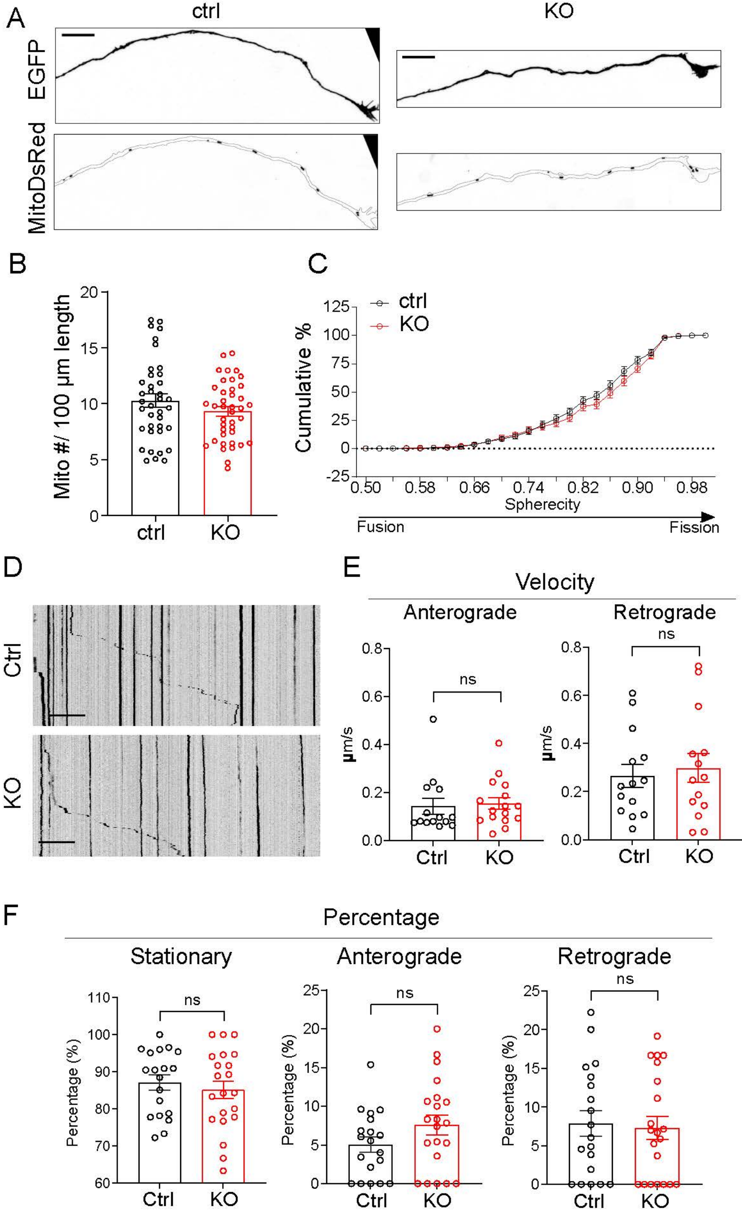

A
ctrl
KO
EGFP
MitoDsRed
B
Mito #/ 100 µm length
ctrl
KO
C
Cumulative %
ctrl
KO
Sphercity
Fusion
Fission
D
Ctrl
KO
E
Velocity
Anterograde
Retrograde
µm/s
ns
Ctrl
KO
F
Percentage
Stationary
Anterograde
Retrograde
Percentage (%)
ns
Ctrl
KO

Fig 5-S1

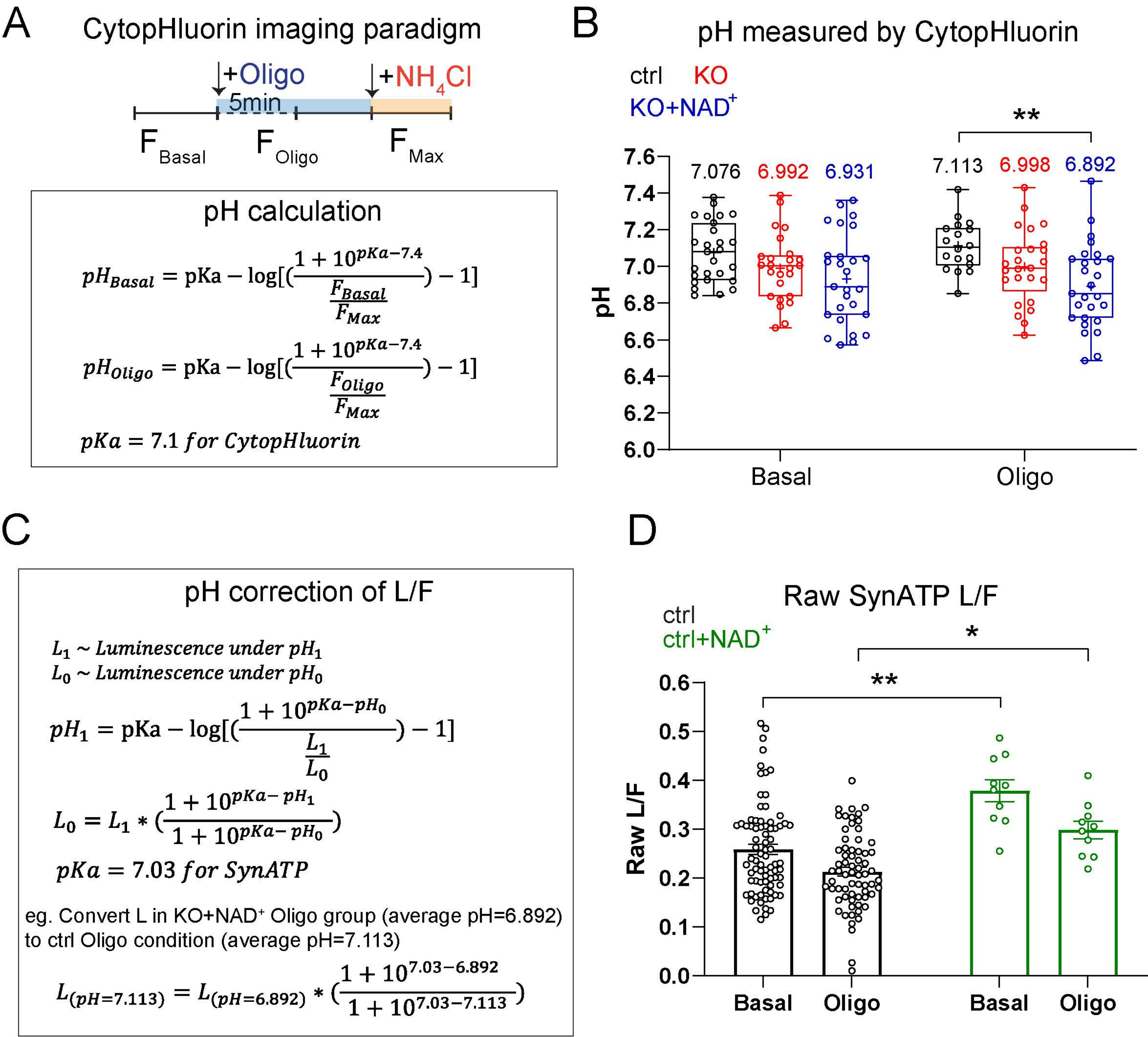
A
CytopHluorin imaging paradigm
+Oligo
+NH_4Cl
5min
F_{Basal}
F_{Oligo}
F_{Max}
pH calculation
$pH_{Basal} = \mathrm{pKa} - \log[(\frac{1 + 10^{pKa-7.4}}{\frac{F_{Basal}}{F_{Max}}}) - 1]$
$pH_{Oligo} = \mathrm{pKa} - \log[(\frac{1 + 10^{pKa-7.4}}{\frac{F_{Oligo}}{F_{Max}}}) - 1]$
$pKa = 7.1\ for\ CytopHluorin$
B
pH measured by CytopHluorin
ctrl
KO
KO+NAD^+
**
7.076
6.992
6.931
7.113
6.998
6.892
pH
7.6
7.4
7.2
7.0
6.8
6.6
6.4
6.2
6.0
Basal
Oligo
C
pH correction of L/F
$L_1 \sim Luminescence\ under\ pH_1$
$L_0 \sim Luminescence\ under\ pH_0$
$pH_1 = \mathrm{pKa} - \log[(\frac{1 + 10^{pKa-pH_0}}{\frac{L_1}{L_0}}) - 1]$
$L_0 = L_1 * (\frac{1 + 10^{pKa-\ pH_1}}{1 + 10^{pKa-\ pH_0}})$
$pKa = 7.03\ for\ SynATP$
eg. Convert L in KO+NAD^+ Oligo group (average pH=6.892)
to ctrl Oligo condition (average pH=7.113)
$L_{(pH=7.113)} = L_{(pH=6.892)} * (\frac{1 + 10^{7.03-6.892}}{1 + 10^{7.03-7.113}})$
D
Raw SynATP L/F
ctrl
ctrl+NAD^+
*
**
Raw L/F
0.6
0.5
0.4
0.3
0.2
0.1
0.0
Basal
Oligo
Basal
Oligo

Fig 5-S2

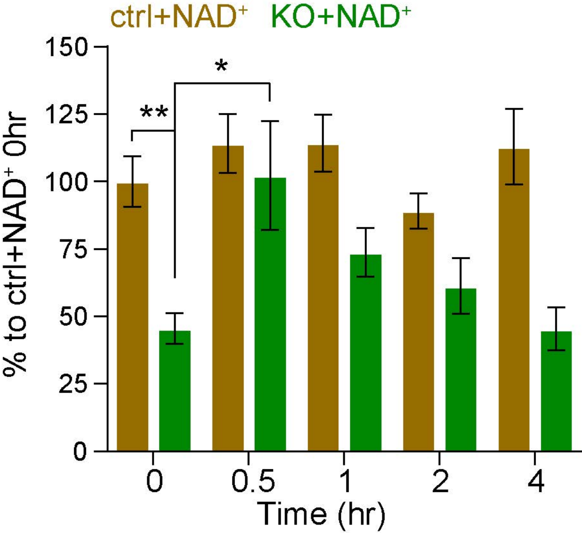

ctrl+NAD+ KO+NAD+
150
125
100
75
50
25
0
% to ctrl+NAD+ 0hr
**
*
0
0.5
1
2
4
Time (hr)

Fig 6-S1

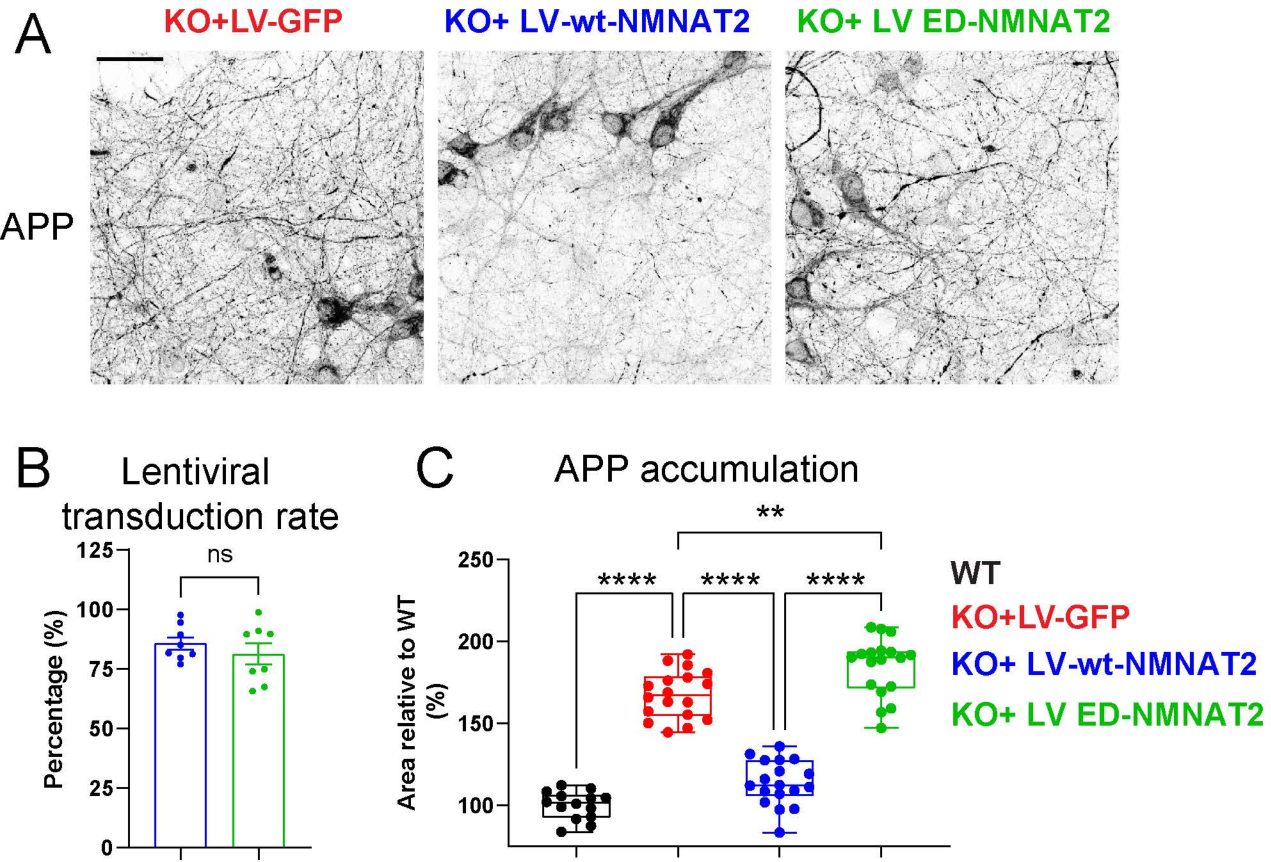
A
KO+LV-GFP
KO+ LV-wt-NMNAT2
KO+ LV ED-NMNAT2
APP
B
Lentiviral transduction rate
ns
Percentage (%)
125
100
75
50
25
0
C
APP accumulation
**
****
****
****
Area relative to WT (%)
250
200
150
100
WT
KO+LV-GFP
KO+ LV-wt-NMNAT2
KO+ LV ED-NMNAT2

Fig 6-S2

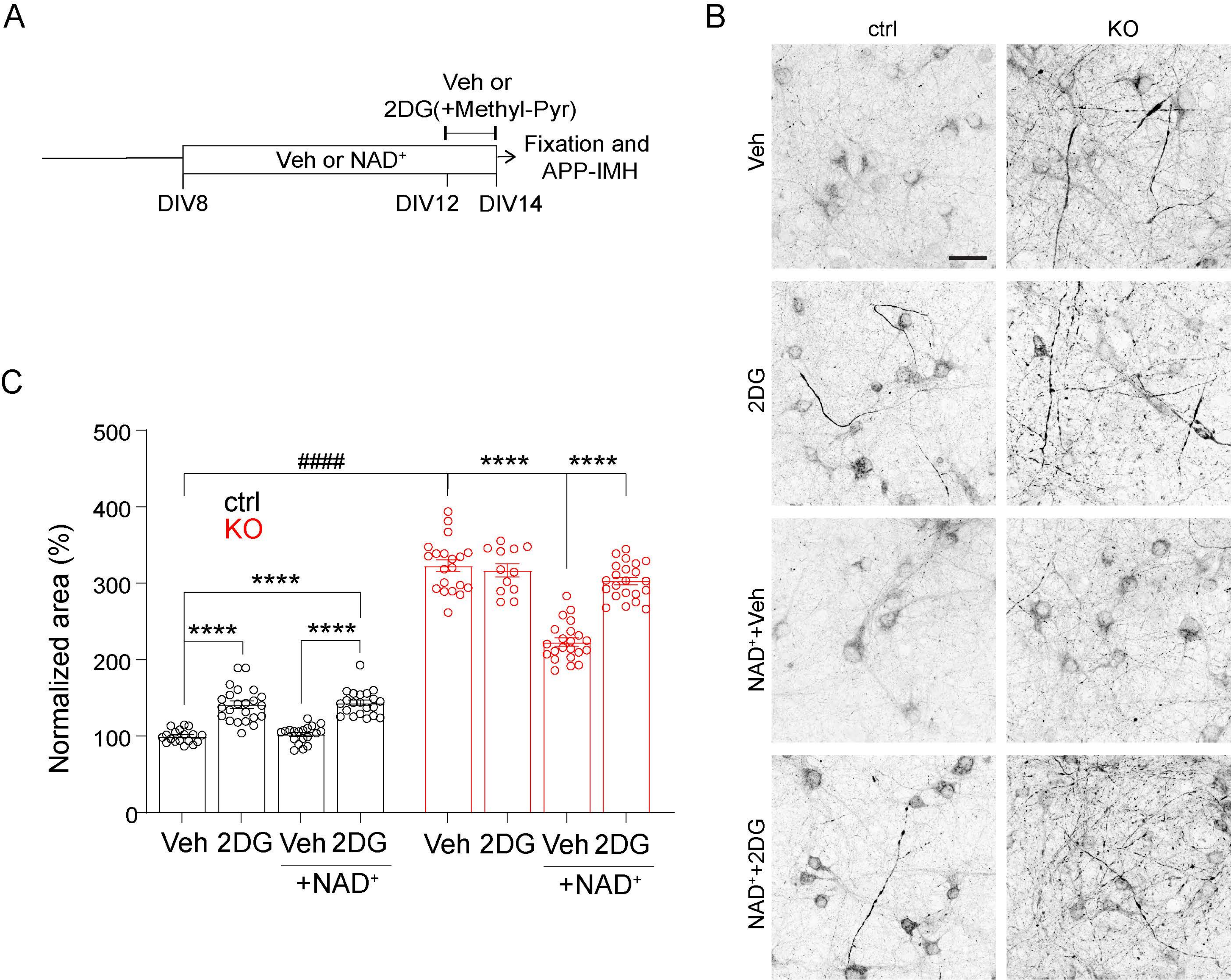
A
Veh or
2DG(+Methyl-Pyr)
Veh or NAD+
Fixation and
APP-IMH
DIV8
DIV12
DIV14
B
ctrl
KO
Veh
2DG
NAD+ +Veh
NAD+ +2DG
C
Normalized area (%)
500
400
300
200
100
0
ctrl
KO
####
****
Veh
2DG
+NAD+

Fig 7-S1

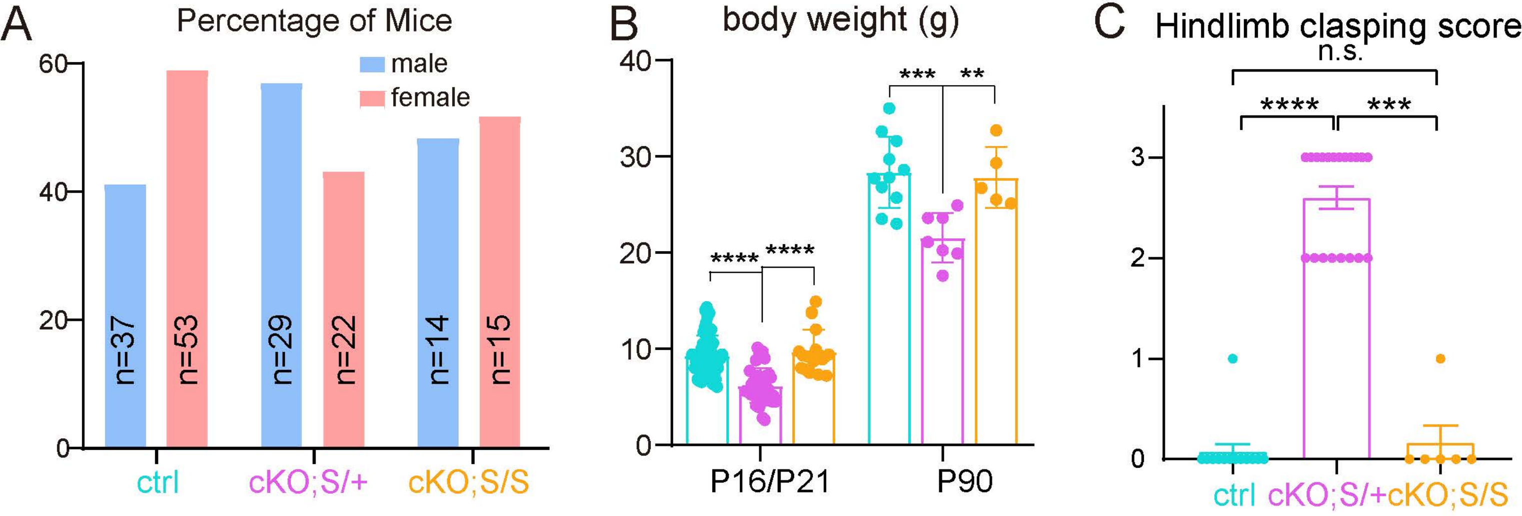
A
Percentage of Mice
male
female
n=37
n=53
n=29
n=22
n=14
n=15
ctrl
cKO;S/+
cKO;S/S
0
20
40
60
B
body weight (g)
****
****
***
**
P16/P21
P90
0
10
20
30
40
C
Hindlimb clasping score
n.s.
****
***
0
1
2
3
ctrl
cKO;S/+
cKO;S/S

Fig 7-S2

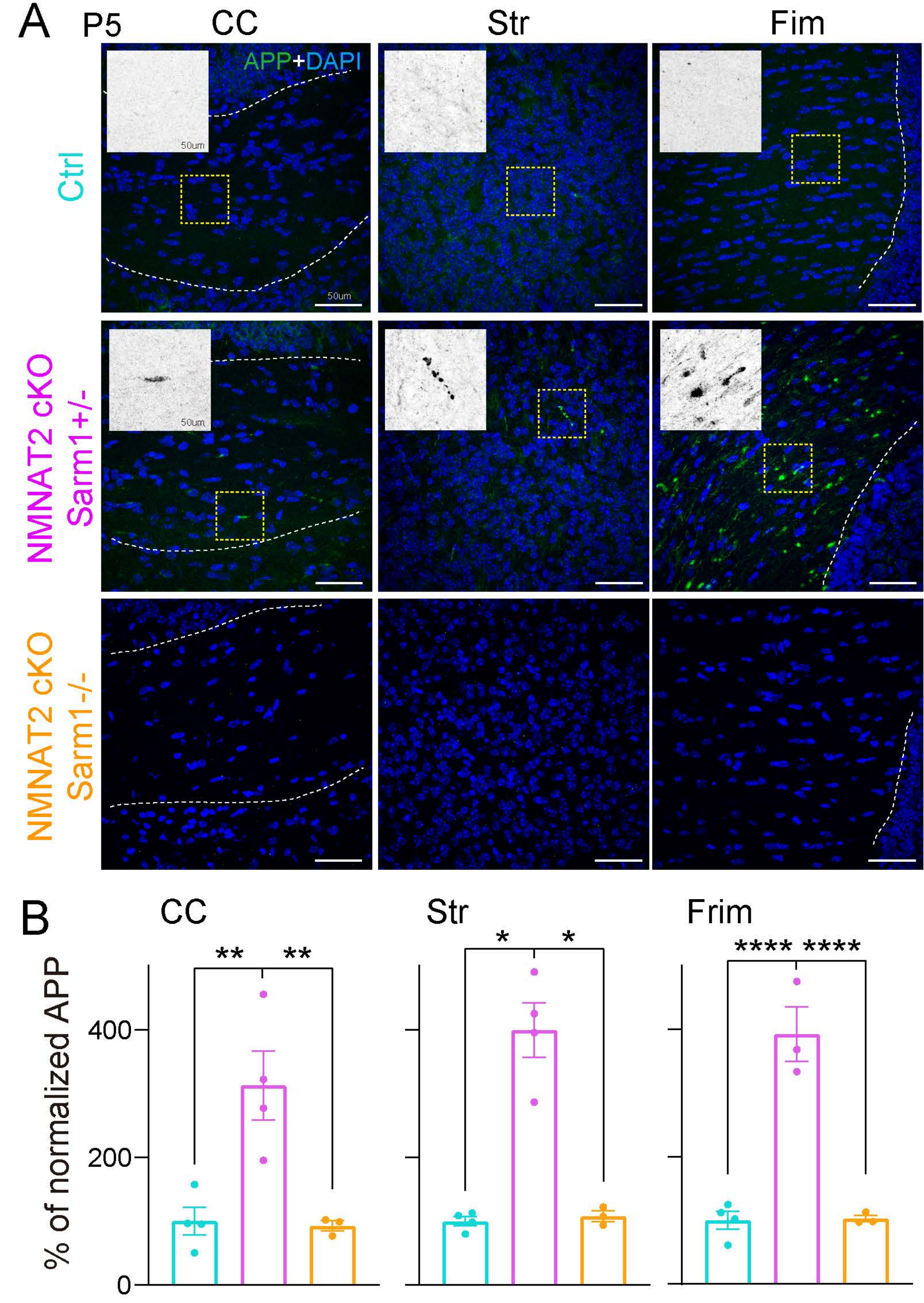
A
P5
CC
Str
Fim
APP+DAPI
50um
Ctrl
NMNAT2 cKO
Sarm1+/-
NMNAT2 cKO
Sarm1-/-
B
CC
Str
Frim
% of normalized APP
400
200
0
**
**
*
*
****
****

Fig 8-S1

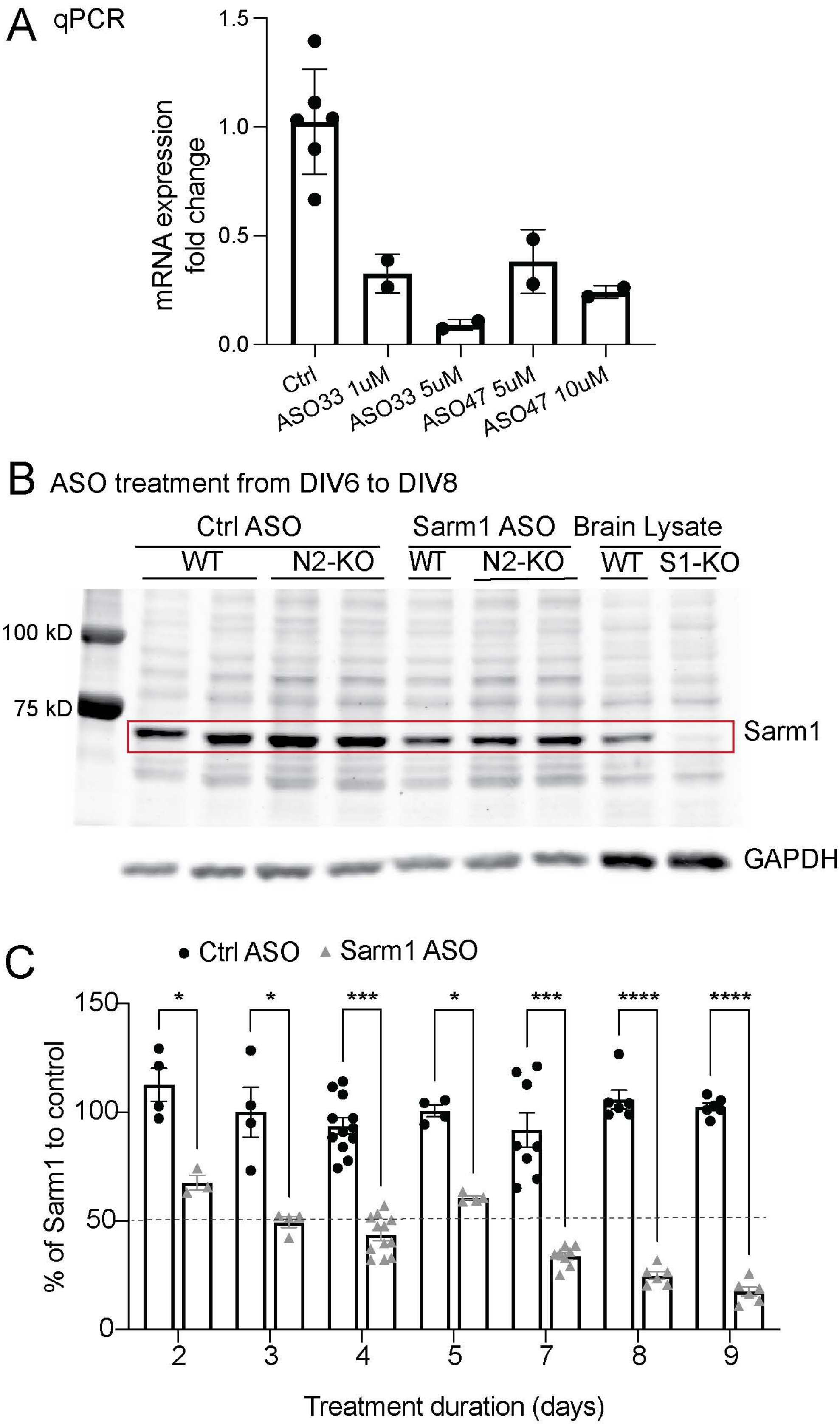
A
qPCR
mRNA expression fold change
1.5
1.0
0.5
0.0
Ctrl
ASO33 1uM
ASO33 5uM
ASO47 5uM
ASO47 10uM
B
ASO treatment from DIV6 to DIV8
Ctrl ASO
Sarm1 ASO
Brain Lysate
WT
N2-KO
WT
N2-KO
WT
S1-KO
100 kD
75 kD
Sarm1
GAPDH
C
Ctrl ASO
Sarm1 ASO
% of Sarm1 to control
150
100
50
0
*
*
***
*
***
****
****
2
3
4
5
7
8
9
Treatment duration (days)

Fig 8-S2

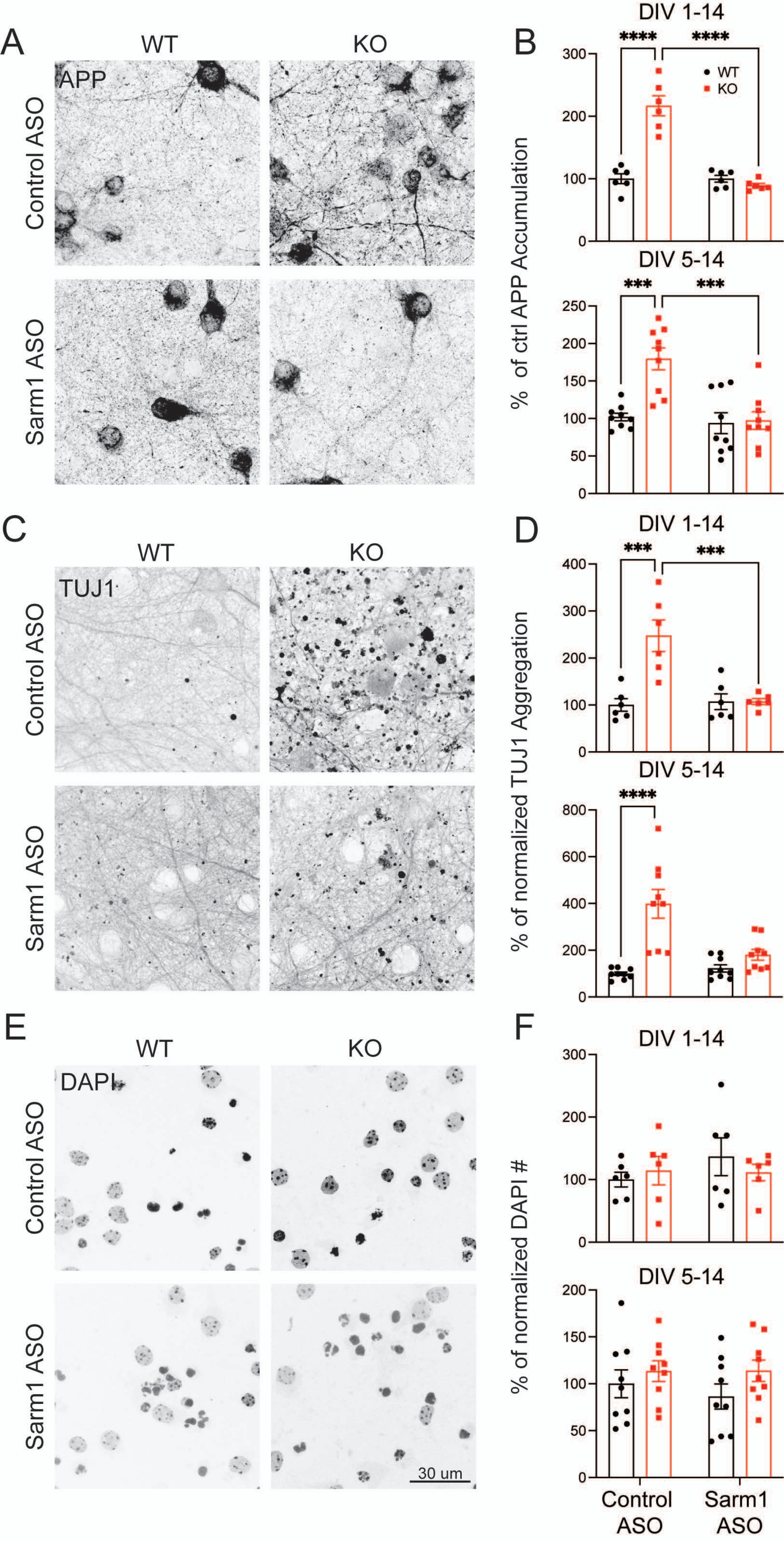
A
WT
KO
APP
Control ASO
Sarm1 ASO
B
DIV 1-14
****
****
WT
KO
300
200
100
0
DIV 5-14
***
***
250
200
150
100
50
0
% of ctrl APP Accumulation
C
WT
KO
TUJ1
Control ASO
Sarm1 ASO
D
DIV 1-14
***
***
400
300
200
100
0
DIV 5-14
****
800
600
400
200
0
% of normalized TUJ1 Aggregation
E
WT
KO
DAPI
Control ASO
Sarm1 ASO
30 um
F
DIV 1-14
300
200
100
0
DIV 5-14
200
150
100
50
0
% of normalized DAPI #
Control ASO
Sarm1 ASO

Fig 8-S3

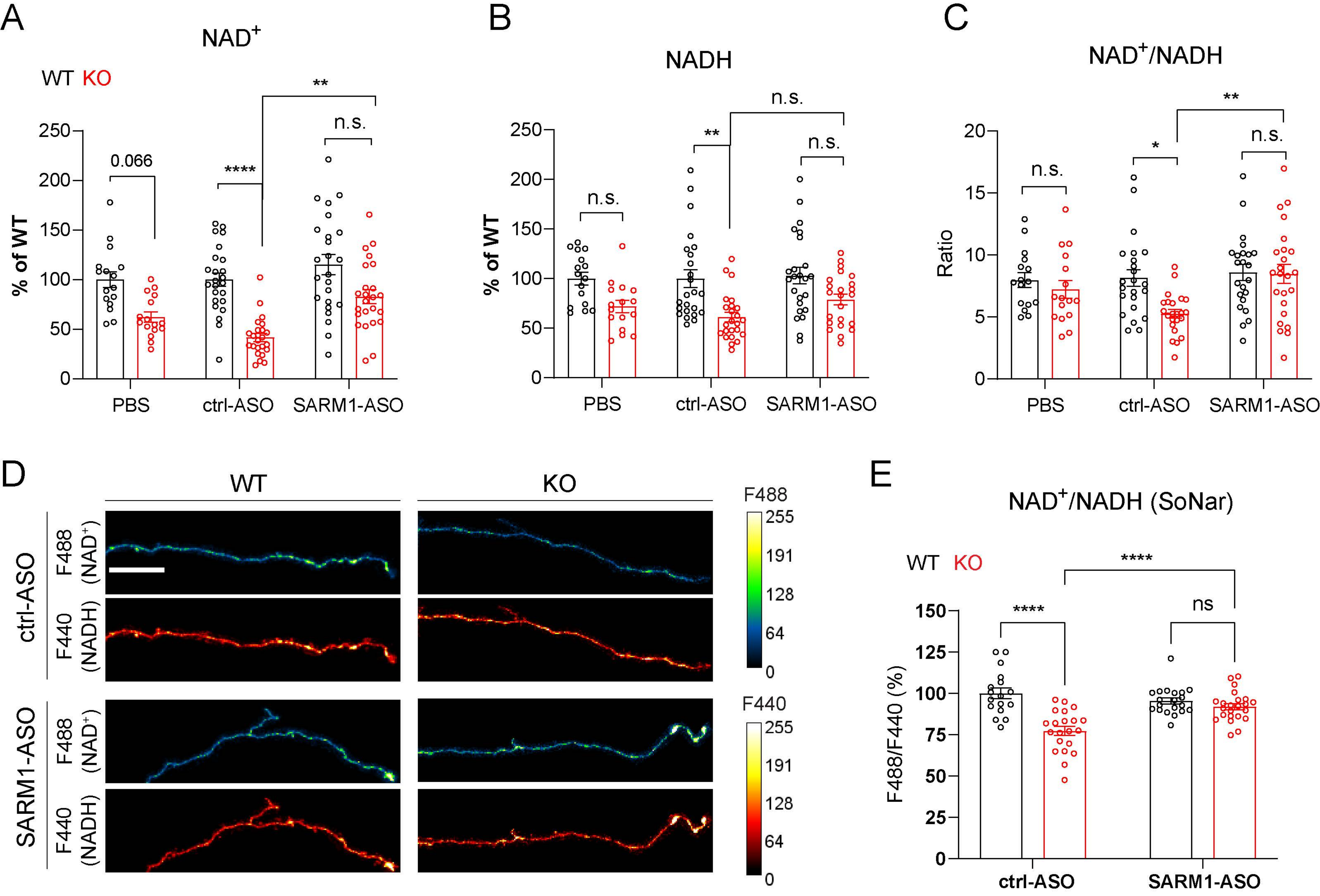
A
NAD+
WT KO
% of WT
250
200
150
100
50
0
0.066
****
**
n.s.
PBS
ctrl-ASO
SARM1-ASO
B
NADH
% of WT
n.s.
**
n.s.
n.s.
PBS
ctrl-ASO
SARM1-ASO
C
NAD+/NADH
Ratio
20
15
10
5
0
n.s.
*
**
n.s.
PBS
ctrl-ASO
SARM1-ASO
D
WT
KO
ctrl-ASO
SARM1-ASO
F488 (NAD+)
F440 (NADH)
F488
255
191
128
64
0
F440
255
191
128
64
0
E
NAD+/NADH (SoNar)
WT KO
F488/F440 (%)
150
125
100
75
50
25
0
****
****
ns
ctrl-ASO
SARM1-ASO
