# Supplementary material for: NMNAT2 supports vesicular glycolysis via NAD homeostasis to fuel fast axonal transport
Source: Mol Neurodegener. 2024 Jan 29;19:13. doi: 10.1186/s13024-023-00690-9 (PMC10823734; doi:10.1186/s13024-023-00690-9)
Supplement: Supplementary file 4 — Additional file 4. [file 13024_2023_690_MOESM4_ESM.docx]

**Sup. Table**

| **Reagents** | **Source** | **Identifier** |
| --- | --- | --- |
| **Antibodies** | | |
| Rabbit monoclonal anti-Amyloid Precursor Protein (APP)  (1:1000 for IHC & ICC) | Abcam | Abcam Cat# ab32136, RRID:AB_2289606 |
| Mouse monoclonal Anti- Myelin Basic Protein (MBP)  (1:1000 for IHC) | Biolegend | BioLegend Cat# 836504, RRID:AB_2616694 |
| Mouse monoclonal Anti-Neurofilament 145 kDa (NFM), CT, clone 3H11  (1:1000 for IHC) | Millipore | Millipore Cat# MAB1621, RRID:AB_94294 |
| Mouse monoclonal Anti-beta-Tubulin III (1:1000 to 1:2000 for ICC) | Millipore | Millipore Cat# 05-559, RRID:AB_309804 |
| Chicken polyclonal anti-MAP2 (1:1000 to 1:2000 for ICC) | Millipore | Millipore Cat# AB5543, RRID: AB_571049 |
| Rabbit polyclonal anti-RFP (1:1000 for ICC) | Rockland | Rockland Cat# 600-401-379, RRID:AB_2209751 |
| Chicken polyclonal anti-GFP (1:1000 for ICC) | Aves Labs | Aves Labs Cat# GFP-1010, RRID:AB_2307313 |
| Goat anti-Mouse IgG (H+L), Alexa Fluor 488 Conjugated (1:1000 for IHC & ICC) | Molecular Probes | Molecular Probes Cat# A-11029, RRID:AB_138404 |
| Goat anti-Rabbit IgG (H+L), Alexa Fluor 555 Conjugated (1:1000 for IHC & ICC) | Molecular Probes | Molecular Probes Cat# A-21429, RRID:AB_2535850 |
| Goat anti-Chicken IgG (H+L), Alexa Fluor 647 Conjugated (1:1000 for ICC) | Innovative Research | Innovative Research Cat# A21449, RRID:AB_1500594 |
| Goat anti-Chicken IgG (H+L), Alexa Fluor 488 Conjugated (1:1000 for ICC) | Molecular Probes | Molecular Probes Cat# A-11039, RRID:AB_142924 |
| Mouse monoclonal anti-SARM1  (1:1000 for WB) | Homemade | Not applicable |
| Mouse monoclonal anti-GAPDH  (1:2000 for WB) | Millipore | Millipore Cat# MAB374, RRID:AB_2107445 |
| Goat anti-Mouse, IRDye® 800CW Conjugated  (1:5000 for WB) | LI-COR | LI-COR Biosciences Cat# 926-32210, RRID:AB_621842 |
| Goat anti-Mouse, IRDye® 680LT Conjugated  (1:5000 for WB) | LI-COR | LI-COR Biosciences Cat# 827-11080, RRID:AB_10795014 |
| Draq5 | Cell Signaling | 4084L |
| DAPI | Invitrogen | D3571 |
| ProLong™ Gold Antifade Mountant with DAPI | Invitrogen | P36931 |
| Dako Mounting Medium | Agilent Technologies | CS70330-2 |
| **Bacterial and virus strains** | | |
| LV-ELF1alpha-copGFP | Baylor vector core |  |
| LV-ELF1alpha-iCre-IRES-copGFP | Baylor vector core |  |
| LV-ELF1alpha-HA-(wt)NMNAT2-IRES-copGFP | Baylor vector core |  |
| LV-ELF1alpha-HA-(H24D)NMNAT2-IRES-copGFP | Baylor vector core |  |
| **Chemicals, peptides, and recombinant proteins** | | |
| Oligomycin | Calbiochem | 495455 |
| Oligomycin | Sigma-Aldrich | O4876 |
| 2-Deoxy-D-glucose | Sigma-Aldrich | D6134 |
| Methyl pyruvate | ACROS Organics™ | AC127650250 |
| NAD | Roche | NAD100-RO |
| Nicotinamide | ACROS Organics™ | AC128271000 |
| Hibernate E Low Fluorescence | BrainBits | HELF |
| Hibernate E Low Fluorescence-Glucose free | BrainBits | Customized |
| Lipofectamine™ 3000 Transfection Reagent | Thermo Fisher Scientific | L3000008 |
| CombiMag | OZ Biosciences | CM20100 |
| Lipofectamine™ 2000 Transfection Reagent | Thermo Fisher Scientific | 11668030 |
| Papain Dissociation System | Worthington Biochem Co. | LK003150 |
| Proteinase K, recombinant, PCR Grade | Roche | RPROTKSOL-RO |
| EconoTaq® PLUS 2X PCR Master Mix | Lucigen | 95024-006 |
| QIAGEN Fast Cycling PCR Kit | Qiagen | 203743 |
| Poly-D-lysine hydrobromide | Sigma-Aldrich | P6407 |
| B27 supplement | Thermo Fisher Scientific | 17504044 |
| GlutaMAX™ Supplement | Thermo Fisher Scientific | 35050061 |
| Penicillin-Streptomycin (10,000 U/mL) | Thermo Fisher Scientific | 15140122 |
| Neurobasal medium | Thermo Fisher Scientific | 21103049 |
| cOmplete™, Mini, EDTA-free Protease Inhibitor Cocktail | Roche | 11836170001 |
| Phosphatase Inhibitor Cocktail 2 | Sigma-Aldrich | P5726 |
| Phosphatase Inhibitor Cocktail 3 | Sigma-Aldrich | P0044 |
| Dodecyltrimethylammonium bromide (DTAB) | Sigma-Aldrich | D8638 |
| Pierce BCA Protein Assay Kit | Thermo Fisher | 23225 |
| QIAGEN Plasmid Plus Maxi Kit | Qiagen | 12963 |
| RNeasy Mini Kit | Qiagen | 74104 |
| **Critical commercial assays** | | |
| NAD/NADH-Glo™ Assay | Promega | G9071 |
| **Experimental models: Organisms/strains** | | |
| Transgenic mice: NMNAT2^f/f^ | [1] | C57BL/6J background |
| Transgenic mice: NEX-Cre | [2] | C57BL/6 background |
| Transgenic mice: NMNAT2-BLAD | [3] | FVB background |
| Transgenic mice: SARM1 KO | [4] | C57BL/6 background |
| **Oligonucleotides** | | |
| Primer for NMNAT2-BLAD genotyping R3:  5'-CCACTGGGAATGTGATGAAAGAAATAAAAGC-3' | This paper |  |
| Primer for NMNAT2-BLAD genotyping RF:  5'-CTGACGTCTATCTAGAAGTACAC-3' | This paper |  |
| Primer for NMNAT2-BLAD genotyping A:  5'-CCATAAGAATAAATGCAGAATTAC-3' | [3] |  |
| Primer for NMNAT2-BLAD genotyping B:  5'-TGTGCAGTTCAGTGGGTCTT-3' | [3] |  |
| Primer for NMNAT2^f/f^ genotyping A:  5'-GCTGGCCTAGGTGGTGATTTGC-3' | This paper |  |
| Primer for NMNAT2^f/f^ genotyping B:  5'-ACTGGGATGGCACGAGACCCTGC-3' | This paper |  |
| Primer for NMNAT2^f/f^ genotyping C:  5'-AGTCATAGACACTAGACAATCG-3' | This paper |  |
| Primer for NEX-Cre genotyping Cre484:  5'-GCATTTCTGGGGATTGCTTA-3' | This paper |  |
| Primer for NEX-Cre genotyping Cre834:  5'-GTCATCCTTAGCGCCGTAAA-3' | This paper |  |
| Primer for SARM1 KO genotyping Sarm1-common:  5'-GAAATGCATGGAGGGGTTG-3' | This paper |  |
| Primer for SARM1 KO genotyping WT-R:  5'-CCACCAAACGTGTCCAATC-3' | This paper |  |
| Primer for SARM1 KO genotyping Mut-R:  5'-TGTGGTTTCCAAATGTGTCAG-3' | This paper |  |
| Primer for SARM1 mRNA qPCR Forward:  5’-TTTGTCCTGGTGCTGTCTG-3’ | Ionis Pharmaceuticals | RTS35985 |
| Primer for SARM1 mRNA qPCR Reverse:  5’-GCCACTCAAAGCCATCAATG-3’ | Ionis Pharmaceuticals | RTS35985 |
| Probe for SARM1 mRNA detection in qPCR:  5’-ACAATCTCCTTGTGCACCCAGTCC-3’ | Ionis Pharmaceuticals | RTS35985 |
| Non-targeting antisense oligonucleotide (ASO):  5’-CCTATAGGACTATCCAGGAA-3’ | Ionis Pharmaceuticals | 676630 |
| Antisense oligonucleotide (ASO47) targeting SARM1:  5’-CCACCTTTTAGTCAAGACCC-3’ | Ionis Pharmaceuticals | 899947 |
| Antisense oligonucleotide (ASO33) targeting SARM1:  5’-GGTAAGAGCCTTAGGCACGC-3’ | Ionis Pharmaceuticals | 899933 |
| **Recombinant DNA** | | |
| pEGFP-n1-APP | [6] | Addgene Plasmid #69924 |
| pEGFP-c1-SNAP25 | [7] |  |
| pEGFP-n1-SYPH (Synaptophysin) | [7] |  |
| pmCherry-n1-NMNAT2 | [7] |  |
| pLV-mitoDsRed | [9] | Addgene plasmid # 44386 |
| pCX-EGFP | [8] |  |
| pcDNA3.1- SoNar | [10] |  |
| pcDNA3.1-cpYFP | [10] |  |
| pCAG-mCherry | Gift from Ken Mackie |  |
| pCMV-MitoVenus | [11] |  |
| pcDNA3-Syn-ATP | [12] | Addgene plasmid #51819 |
| pcDNA3-Cyto-pHluorin | [12] |  |
| **Software and algorithms** | | |
| Fiji (Image J with updated plug-in) | NIH | https://fiji.sc/ |
| NIS-Elements | Nikon | https://www.microscope.healthcare.nikon.com/products/software/nis-elements |
| Imaris | OXFORD | https://imaris.oxinst.com/ |
| GraphPad Prism 9.0 | GraphPad | https://www.graphpad.com/ |
| SPSS | IBM | https://www.ibm.com/analytics/spss-statistics-software |
| **Other** | | |
| Round cover glass, #1.5 thickness, 12 mm | Thomas Scientific | 1217N79 |
| 35 mm Dish \| No. 1.5 Coverslip \| 20 mm Glass Diameter \| Uncoated | MaTek | P35G-1.5-20-C |
| 35 mm Dish \| No. 1.5 Coverslip \| 14 mm Glass Diameter \| Uncoated | MaTek | P35G-1.5-14-C |

1. Gilley J, Adalbert R, Yu G, Coleman MP: **Rescue of peripheral and CNS axon defects in mice lacking NMNAT2.** *J Neurosci* 2013, **33:**13410-13424.

2. Goebbels S, Bormuth I, Bode U, Hermanson O, Schwab MH, Nave KA: **Genetic targeting of principal neurons in neocortex and hippocampus of NEX-Cre mice.** *Genesis* 2006, **44:**611-621.

3. Hicks AN, Lorenzetti D, Gilley J, Lu B, Andersson KE, Miligan C, Overbeek PA, Oppenheim R, Bishop CE: **Nicotinamide mononucleotide adenylyltransferase 2 (Nmnat2) regulates axon integrity in the mouse embryo.** *PLoS One* 2012, **7:**e47869.

4. Kim Y, Zhou P, Qian L, Chuang JZ, Lee J, Li C, Iadecola C, Nathan C, Ding A: **MyD88-5 links mitochondria, microtubules, and JNK3 in neurons and regulates neuronal survival.** *J Exp Med* 2007, **204:**2063-2074.

5. Yaylaoglu MB, Titmus A, Visel A, Alvarez-Bolado G, Thaller C, Eichele G: **Comprehensive expression atlas of fibroblast growth factors and their receptors generated by a novel robotic in situ hybridization platform.** *Dev Dyn* 2005, **234:**371-386.

6. Currinn H, Guscott B, Balklava Z, Rothnie A, Wassmer T: **APP controls the formation of PI(3,5)P(2) vesicles through its binding of the PIKfyve complex.** *Cell Mol Life Sci* 2016, **73:**393-408.

7. Milde S, Gilley J, Coleman MP: **Subcellular localization determines the stability and axon protective capacity of axon survival factor Nmnat2.** *PLoS Biol* 2013, **11:**e1001539.

8. Galiano MR, Jha S, Ho TS, Zhang C, Ogawa Y, Chang KJ, Stankewich MC, Mohler PJ, Rasband MN: **A distal axonal cytoskeleton forms an intra-axonal boundary that controls axon initial segment assembly.** *Cell* 2012, **149:**1125-1139.

9. Kitay BM, McCormack R, Wang Y, Tsoulfas P, Zhai RG: **Mislocalization of neuronal mitochondria reveals regulation of Wallerian degeneration and NMNAT/WLD(S)-mediated axon protection independent of axonal mitochondria.** *Hum Mol Genet* 2013, **22:**1601-1614.

10. Zhao Y, Hu Q, Cheng F, Su N, Wang A, Zou Y, Hu H, Chen X, Zhou HM, Huang X, et al: **SoNar, a Highly Responsive NAD+/NADH Sensor, Allows High-Throughput Metabolic Screening of Anti-tumor Agents.** *Cell Metab* 2015, **21:**777-789.

11. Cambronne XA, Stewart ML, Kim D, Jones-Brunette AM, Morgan RK, Farrens DL, Cohen MS, Goodman RH: **Biosensor reveals multiple sources for mitochondrial NAD(+).** *Science* 2016, **352:**1474-1477.

12. Rangaraju V, Calloway N, Ryan TA: **Activity-driven local ATP synthesis is required for synaptic function.** *Cell* 2014, **156:**825-835.

13. Fletcher TL, Cameron P, De Camilli P, Banker G: **The distribution of synapsin I and synaptophysin in hippocampal neurons developing in culture.** *J Neurosci* 1991, **11:**1617-1626.

14. Chen CY, Lin CW, Chang CY, Jiang ST, Hsueh YP: **Sarm1, a negative regulator of innate immunity, interacts with syndecan-2 and regulates neuronal morphology.** *J Cell Biol* 2011, **193:**769-784.

15. Clements RT, Fuller LE, Kraemer KR, Radomski SA, Hunter-Chang S, Hall WC, Kalantar AA, Kraemer BR: **Quantification of Neurite Degeneration with Enhanced Accuracy and Efficiency in an In Vitro Model of Parkinson's Disease.** *eNeuro* 2022, **9**.
